# On parametric representation of the Newton's aerodynamic problem

**DOI:** 10.1016/j.heliyon.2023.e16721

**Published:** 2023-05-29

**Authors:** Alexandr A. Barsuk, Florentin Paladi

**Affiliations:** Department of Theoretical Physics and ePhysMCS Lab, Faculty of Physics and Engineering, Moldova State University, A. Mateevici Str. 60, Chisinau MD-2009, Republic of Moldova

**Keywords:** Newton's aerodynamic problem, Isoperimetric variational problem, Solid of revolution, Parametric representation

## Abstract

Newton's problem of finding the surface shape of a rotation body based on the condition of minimal resistance of the body when it moves in a rarefied medium is discussed. The problem is formulated in the form of a classical isoperimetric problem in calculus of variations. The exact solution is given in the class of piecewise differentiable functions. The numerical results of specific calculations of the functional for cone and hemisphere are presented. We prove that the optimization effect is significant by comparison of the results for cone and hemisphere with the value of the optimized functional for the optimal contour.

## 1 Introduction

The problem of finding the resistance of an axisymmetric body moving in a rarefied medium was first analyzed by Sir Isaac Newton in his “Mathematical Principles of Natural Philosophy”. Newton published the solutions for specific calculations of the resistance of the axisymmetric bodies of a given shape. Also, Newton's minimal resistance problem, i.e., the problem of determining the shape of the surface of a solid of revolution, which experiences a minimum resistance when it moves through a homogeneous fluid with constant velocity in the direction of the axis of revolution, was studied in 1685 [1]. This is the first example of a problem solved in what is now called the calculus of variations, appearing a decade before the brachistochrone problem (see, for example, Ref. [2]).

**Fig. 1:**
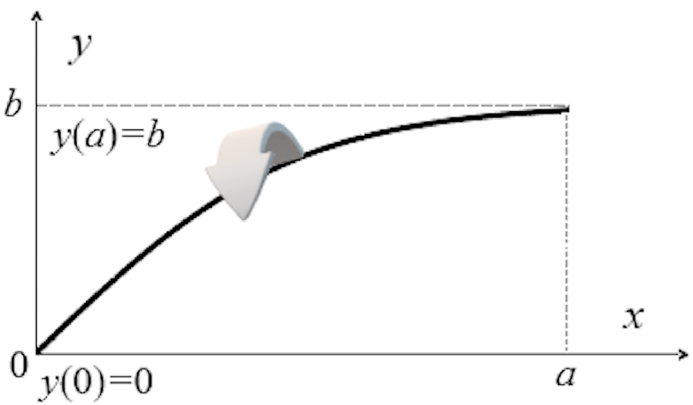
Generation of a convex surface by the function *y*(*x*).

Let us proceed to the mathematical formulation of the Newton's problem of a body of minimal aerodynamic resistance, or Newton's aerodynamic problem, in terms of the resistance to the movement of a body in an ideally elastic medium of particles of low density, that is

(1) $$J\left[y\right]=\int_{0}^{a}\frac{xdx}{1+y^{\prime }{\left(x\right)}^{2}}\to \min_{y\left(x\right)},y\left(0\right)=0,y\left(a\right)=b$$

where the integrand in Eq. (1) is always positive; then the values of functional J[y]≥0 for arbitrary dependences *y*(*x*) for the specified boundary conditions at *x* = 0 and *x* = *a*. The profile is a surface of revolution of a curve *y*(*x*) which generates a solid body shape when it is rotated about the *x*-axis as it is shown by arrow in Fig. 1 in the case of a convex surface, i.e., under the additional conditions that the body is convex and rotationally symmetric.

For an explicit expression of the function *y*(*x*) satisfying the boundary conditions in Eq. (1), one can consider

$$y_{n}\left(x\right)=\frac{b}{a}x+\sin^{2}\frac{n\pi }{a}x$$

where *n* is an integer. One can note that $\lim_{n\to \infty }J\left[y_{n}\right]=0$ and, thus, the functional $J\left[y_{n}\right]$ reaches its smallest value on contours $y_{n}\left(x\right)$ that are non-differentiable in the interval [0, *a*] (see, for example, Ref. [3]). Thereby, in order to regularize the solutions of variational problem in the formulation of Eq. (1), it was proposed to look for a solution in the class of monotone increasing dependences *y*(*x*), i.e., satisfying the condition $y^{\prime }\left(x\right)\geq 0$. From physics perspective, if the space is filled with a uniformly distributed rarefied fluid, it is assumed that every molecule of the fluid hits the body only once; therefore, it is necessary to introduce a condition that the function *y*(*x*), whose graph generates the rotating body shape like in Fig. 1, is monotone increasing. As a result, we arrive at the formulation of the Newton's problem in the form

(2) $$J\left[y\right]=\int_{0}^{a}\frac{xdx}{1+y^{\prime }{\left(x\right)}^{2}}\to \min_{y\left(x\right)},y^{\prime }\left(x\right)\geq 0,y\left(0\right)=0,y\left(a\right)=b$$

Eq. (2) includes unilateral constraints in the form of coupled differential equations, which make impossible to solve it by using classical algorithms for solving variational problems. A detailed analysis of the solution of problem in the formulation of Eq. (2) by transforming it to an optimal control problem and solving it by using methods for solving optimal control problems is given in Ref. [4], [page 43]. As a result of such analysis, it is shown that the solution of this variational problem is function *y*(*x*), which has one corner point at $x=x_{*}$, wherein

$$y\left(x\right)\equiv 0,0\leq x\leq x_{*},y^{\prime }\left(x\right)\geq 1,x_{*}\leq x\leq a$$

One can note various methods of investigating the Newton's aerodynamic problem considering nonsymmetric cases within the framework of the Newtonian sine-squared pressure law [5], and an extension to the Newton solution on the shape of the profile of an axisymmetric rigid body which, under prescribed length and caliber, has the least aerodynamic resistance in the presence of friction [6], as well as the existence of a body of minimal resistance with prescribed volume was proved in the class of profiles which satisfy a two sided bound on the mean curvature [7]. Also, the Newton's problem was generalized to the bodies which are not rotating, to determine a convex body of the given length and of the given maximal cross section, which has the minimal resistance when moving in the rarefied medium [8]. The Newton's aerodynamic problem of finding the local properties of convex surfaces near ridge point was solved in the case of convex bodies [9], while giving insights into the structure of singular points of a solution to Newton's least resistance problem [10]. We also note Ref. [11] in which, from a unified point of view, the well-known classical solutions of the Newton's aerodynamic problem are discussed using the Legendre transform and Hessian measures.

We analyze in this paper the variational problem in the formulation of Eq. (2), which is transformed into an equivalent isoperimetric variational problem, and, in such a way, we arrive at the parametric representation for the Newton's aerodynamic problem for the solution of which classical methods for solving variational problems can already be applied. For convenience and completeness of the presentation in Section 2, a detailed transformation of the Newton's problem to an isoperimetric variational problem is given and the necessary conditions for an extremum are formulated. Parametric representation and solutions for the Newton's aerodynamic problem, including their asymptotic expressions are analyzed in Section 3. Based on these analytical results and numerical data obtained in Section 4, we formulate the main conclusions in Section 5.

## 2 Transformation of the Newton's problem to an isoperimetric variational problem

### 2.1 Formulation of the isoperimetric variational problem

To remove the unilateral constraints in Eq. (2), we introduce a real function *u*(*x*) according to the condition

(3) $$y^{\prime }\left(x\right)=u^{2}\left(x\right)$$

and, thus, the unilateral constraints $y^{\prime }\left(x\right)\geq 0$ are satisfied for arbitrary dependencies *u*(*x*). After integrating Eq. (3) in the interval [0,x] and considering the boundary condition y(0)=0, the function *y*(*x*) can be represented as

(4) $$y\left(x\right)=\int_{0}^{x}u^{2}\left(s\right)ds$$

where 0≤x≤a, and the boundary condition at *x* = *a* can be expressed accordingly by the equation

(5) $$y\left(a\right)=\int_{0}^{a}u^{2}\left(s\right)ds=b$$

Therefore, constant *b* is the upper limit of the function *y*(*x*) as defined by Eq. (1) and depicted in Fig. 1.

As a result, the variational problem (3) can be formulated with respect to the function *u*(*x*) as an isoperimetric variational problem, that is

(6) $$J\left[u\right]=\int_{0}^{a}\frac{xdx}{1+u^{4}\left(x\right)}\to \min_{u\left(x\right)},I\left[u\right]=\int_{0}^{a}u^{2}\left(x\right)dx=b$$

Note that the functionals J[u],I[u] do not contain derivatives $u^{\prime }\left(x\right)$, which leads to significant simplification in analysis of the isoperimetric variational problem (6).

### 2.2 Necessary conditions for an extremum

Let us further use the method of indefinite Lagrange multipliers to obtain the necessary conditions for an extremum in the problem under discussion, according to which we introduce the extended Lagrange functional

Λ[u]=J[u]−λ(I[u]−b)

where λ is Lagrange multiplier, and one can proceed to the mathematical formulation of the variational problem

(7) $$\Lambda \left[u\right]=J\left[u\right]-\lambda \left(I\left[u\right]-b\right)\to \min_{u\left(x\right)}$$

For convenience of analysis, let us represent the Lagrange functional in the form

(8) $$\Lambda \left[u\right]=\int_{0}^{a}F\left(x,u\right)dx,\;F\left(x,u\right)\equiv \frac{x}{1+u^{4}\left(x\right)}-\lambda \left(u^{2}\left(x\right)-b\right).$$

Using standard calculations in the expression for the first variation of functional (8), that is

$$\delta \Lambda \left[u\right]=\int_{0}^{a}\delta F\left(x,u\right)dx=\int_{0}^{a}\left(\partial F/\partial u\right)\delta udx$$

we arrive at the *necessary condition for an extremum*, i.e., the variation term vanishes

$$\left(\frac{2xu^{2}}{{\left(1+u^{4}\right)}^{2}}+\lambda \right)u\equiv 0$$

Thus, the extremals of the variational problem (6) are given by function *u*(*x*) defined by the relations

(9) $$u\left(x\right)\equiv 0,\frac{2xu^{2}}{{\left(1+u^{4}\right)}^{2}}+\lambda \equiv 0$$

First of all, we note that the implementation of each of the solutions (9) on the entire interval [0,a] is not possible, which means that there are no solutions to the problem (6) in the class of continuous functions *u*(*x*) in the interval [0,a], and this, in turn, leads to the conclusion that the solutions *y*(*x*) in the interval [0,a] are not smooth and necessarily have corner points. Indeed, we have I[u]=0≠b in the case of u(x)≡0 and, thus, the isoperimetric condition I[u]=b is not satisfied. Therefore, the implementation of this solution on the entire interval [0,a] is not possible.

Regarding the possibility of realizing the second solution in Eq. (9) on the entire interval [0,a], we note that it is necessary that the Lagrange multiplier λ be nonzero (λ≠0) and negative. Therefore, we introduce a positive constant c>0 according to the equality λ=−c, and rewrite the second of the identities in Eq. (9) in the form

(10) $$u^{2}=\frac{c}{2}\frac{{\left(1+u^{4}\right)}^{2}}{x}$$

Integrating both parts of Eq. (10) in the interval [0,a] and, taking into account the integral constraint in Eq. (6), we arrive at the equality

(11) $$\int_{0}^{a}u^{2}dx=b=\frac{c}{2}\int_{0}^{a}\frac{{\left(1+u^{4}\right)}^{2}}{x}dx$$

the fulfillment of which is not possible due to the divergence of the last integral in Eq. (11).

As a result, we conclude that the solution of variational problem (6) is given in the class of piecewise continuous functions, while on each of the continuity intervals for u(x) the solutions are described by relations represented in a convenient form for the subsequent analysis and which are equivalent to Eq. (9), that is

(12) $$u\left(x\right)\equiv 0,x=\frac{c}{2}\frac{{\left(1+u^{4}\right)}^{2}}{u^{2}}$$

The structure of the Lagrange function in Eq. (7) and the *necessary extremum conditions* (12) allow us to draw an important conclusion about the properties of solutions u(x) for the variational problem described by Eqs. (6), (7).

### 2.3 Properties of solutions and relations between parameters

Let u(x)<0 on a certain interval $x_{1}\leq x\leq x_{2}$. Since u(x) has an even power in all relations (7)–(12), when u(x) is replaced on this interval by −u(x), then all Eqs. (7), (8), (9), (10), (11), (12), including the value of functional in Eq. (7), do not change. This property allows us to make significant simplifications in the subsequent analysis and representation of the variational problem solutions. Taking this remark into account, we solve the variational problem for the class of non-negative functions, i.e., u(x)≥0, without of any loss of generality in the analysis, and thus one can further consider

(13) $$u\left(x\right)\geq 0,\;0\leq x\leq a.$$

Let us denote by $x_{*}$ the coordinate of discontinuity points of the function u(x) or one of the points in the case of the existence of several or even an infinite number of discontinuity points. Let us also introduce the notations $u_{-}=\lim_{\varepsilon \to 0>0}u\left(x_{*}-\varepsilon \right)$ for the left-hand and $u_{+}=\lim_{\varepsilon \to 0>0}u\left(x_{*}+\varepsilon \right)$ for the right-hand values of the solution u(x) at the discontinuity point (in this case $u_{+}\neq u_{-}$), and let us formulate the conjugation condition for solutions at the discontinuity point. For this purpose, we use the expression for the first variation of the extended functional, due to the variation of the discontinuity point $x_{*}$. We have $\delta \Lambda \left[u\right]=\left(F\left(x_{*},u_{-}\right)-F\left(x_{*},u_{+}\right)\right)\;\delta x_{*}$ and, based on the condition that this variation vanishes, we obtain the conjugation condition for solutions at the discontinuity point $F\left(x_{*},u_{-}\right)=F\left(x_{*},u_{+}\right)$, which takes the form

(14) $$\frac{x_{*}}{1+u_{-}^{4}}-\lambda u_{-}^{2}=\frac{x_{*}}{1+u_{+}^{4}}-\lambda u_{+}^{2}$$

where the definition of F(x,u) in Eq. (8) is taking into account. Using standard calculations in Eq. (14) and considering the expressions $u_{+}\neq u_{-}$ and λ=−c, one can write

(15) $$\frac{x_{*}\left(u_{+}^{2}+u_{-}^{2}\right)}{\left(1+u_{+}^{4}\right)\left(1+u_{-}^{4}\right)}=c$$

Using Eq. (12) and taking into account Eq. (15) for $u_{+}\neq 0$ and $u_{-}\neq 0$, we arrive at two additional relations for the parameters $x_{*}$, $u_{+},u_{-},c$, that is

(16) $$x_{*}=\frac{c}{2}\frac{{\left(1+u_{+}^{4}\right)}^{2}}{u_{+}^{2}},x_{*}=\frac{c}{2}\frac{{\left(1+u_{-}^{4}\right)}^{2}}{u_{-}^{2}}$$

Considering Eq. (16) and the fulfillment of condition $u_{\pm }>0$ (see Eq. (13)), after excluding parameters $x_{*}$ and c, we arrive at the relation

(17) $$u_{+}u_{-}\left(u_{+}^{2}+u_{+}u_{-}+u_{-}^{2}\right)=1$$

In particular, if one of the parameters *u*_+_, *u*_−_ vanishes, conjugation condition (15) takes the form

(18) $$\frac{x_{*}u_{+}^{2}}{1+u_{+}^{4}}=c\;\left(u_{-}=0\right),\;\frac{x_{*}u_{-}^{2}}{1+u_{-}^{4}}=c\;\left(u_{+}=0\right).$$

Also, considering expressions (16) and (18), and the properties of solutions (13), we arrive at the values

(19) $$u_{+}=1\;\left(u_{-}=0\right),u_{-}=1\;\left(u_{+}=0\right)$$

Thus, in the case $u_{+}\neq 0$ and $u_{-}\neq 0$ the solution parameters $x_{*}$, $u_{+},u_{-}\text{,}$ and c satisfy Eqs. (15), (16) at each discontinuity point of the solutions for the variational problem under discussion. When one of the parameters $u_{+}$, $u_{-}$ vanishes, the corresponding values of parameters are given by Eq. (19) and, in accordance with Eq. (18), we arrive at an expression for the coordinate of the discontinuity point of the solution

(20) $$x_{*}=2c$$

One can note that, in accordance with Eq. (20) for the cases under consideration, there is a single discontinuity point of the solution with the coordinate determined by this expression. The only admissible solutions of the variational problem are characterized by the values of solutions at discontinuity points for which $u_{-}=0$, $u_{+}\neq 0$ and $u_{-}\neq 0$, $u_{+}=0$ (see Eq. (19)). However, for this class of solutions, the coordinates of the discontinuity points are determined by a single expression (20), and thus the solution of the variational problem (6) has only one discontinuity point.

Let us now analyze the possibility of obtaining the solution of variational problem with a discontinuity point at which $u_{+}\neq u_{-}\neq 0$. In this case, four parameters $x_{*}$, $u_{+}\text{,}$ $u_{-}\text{,}$ and c satisfy conditions defined by Eqs. (15), (16). For convenience of the subsequent analysis, we introduce notation $\gamma =u_{-}/u_{+}$ and, taking into account Eq. (17), one can conclude that the following parametric representations for the values of $u_{+},u_{-}$ can be further used in solving the problem

(21) $$u_{+}^{4}=\frac{1}{\gamma \left(1+\gamma +\gamma^{2}\right)},\;u_{-}^{4}=\frac{\gamma^{3}}{1+\gamma +\gamma^{2}}\;\left(\gamma >0\right).$$

Let us analyze in more detail Eqs. (15), (16). We exclude the parameters $x_{*}$ and c in these equations, and arrive at the relation

$$\frac{u_{+}^{2}+u_{-}^{2}}{1+u_{-}^{4}}=\frac{2u_{+}^{2}}{1+u_{+}^{4}}$$

In addition, taking into account Eq. (21), the equation with respect to the parameter γ can be represented as $1+2\gamma^{2}=\gamma +\gamma^{2}+\gamma^{3}$ or in its final form

(22) $$\left(1-\gamma \right)\left(1+\gamma^{2}\right)=0$$

A similar analysis of Eq. (15) and the second of relations in Eq. (16) also leads to Eq. (22) with respect to the parameter γ. Thus, the fulfillment of Eqs. (15), (16) is possible only if the value of parameter γ=1, i.e., for $u_{-}=u_{+}$, which corresponds to the case of continuous solutions. Therefore, solutions of this variational problem with discontinuity points at which $u_{-}\neq 0$, $u_{+}\neq 0$, and $u_{+}\neq u_{-}$ cannot be obtained.

One can also note that the interval section for which the solution u(x)≡0 is adjacent to the left end of the interval [0,a]. As a result, the solution of variational problem (15) is described by the relations

(23) $$u\left(x\right)\equiv 0\;\left(0\leq x\leq x_{*}\right),x=\frac{c}{2}\frac{{\left(1+u^{4}\right)}^{2}}{u^{2}}\;\left(x_{*}\leq x\leq a\right)$$

while $u_{+}=u\left(x_{*}\right)=1$ and the parameter value c is determined from the isoperimetric condition I[u]=b.

### 2.4 Second variation of the optimized functional

At the end of this section, let us proceed to the analysis of the extremum reached by the functional of the variational problem in the formulation of Eq. (8) on the given solutions. In this regard, we calculate the second variation of the optimized functional and represent it in the form

$$\delta^{2}\Lambda \left[u\right]=\int_{0}^{a}\delta^{2}F\left(x,u\right)dx=\int_{0}^{a}{\left(\partial^{2}F/\partial u^{2}\right)\left(\delta u\right)}^{2}dx$$

Considering the definition of F(x,u) in Eq. (8), the expression for the Lagrange multiplier λ in Eq. (9), and the representation of the optimal solution by Eq. (23), we arrive at a representation for the second variation in the form

$$\delta^{2}\Lambda \left[u\right]=8\int_{x_{*}}^{a}\frac{xu^{2}}{{\left(1+u^{4}\right)}^{3}}{\left(-1+3u^{4}\right)\left(\delta u\right)}^{2}dx$$

In accordance with the above analysis u(x)≥1 for $x\geq x_{*}$. For these values the expression $-1+3u^{4}>0$, and, as a result, we conclude that $\delta^{2}\Lambda \left[u\right]>0$. Thus, the optimized functional in the class of functions under consideration reaches its minimum on the obtained solution of the variational problem.

## 3 Parametric and asymptotic representations and analysis of solutions

First of all, we note that y(x)≡0 in the interval $0\leq x\leq x_{*}$ due to the solutions u(x)≡0 for the variational problem (3) and the representation (4) for y(x). In order to obtain the solution y(x) on the interval $x_{*}\leq x\leq a$, one can use Eq. (3) in the form $dy\left(x\right)=u^{2}dx$, and the corresponding boundary condition defined by Eq. (5). Let us further consider the representation for solution u(x) in Eq. (12) as a parametric assignment of the coordinate $x=\frac{c}{2}\frac{{\left(1+\tau^{2}\right)}^{2}}{\tau }$ ($\tau =u^{2}>0$). Taking this into account, we obtain the expression for the differential $dy=u^{2}dx=\tau dx\left(\tau \right)$, after the integration of which we arrive at the parametric definition of the desired contour y(x), that is

(24) $$x\left(\tau \right)=\frac{c}{2}\left(\frac{1}{\tau }+2\tau +\tau^{3}\right),\;y\left(\tau \right)=\frac{c}{2}\left(\ln\frac{1}{\tau }+\tau^{2}+\frac{3}{4}\tau^{4}\right)+C,\;\tau \geq 1,$$

where *C* is the integration constant. Note that the value $x=x_{*}=2c$ corresponds to the value of parameter $\tau =\tau_{+}=1$ and, as a result, we have $x^{\prime }\left(\tau \right)>0$ at $\tau >\tau_{+}$. Indeed, $x^{\prime }\left(\tau \right)=0$ at $\tau =\tau_{*}=1/3$ and for the interval $\tau >\tau_{+}>\tau_{*}$ we have $x^{\prime }\left(\tau \right)>0$. Therefore, as the parameter τ increases, the function x(τ) monotone increases to its limiting value x=a at $\tau =\tau_{1}$. On the other hand, we have $y\left(x_{*}\right)=y\left(\tau_{+}\right)=0$ at $x=x_{*}$, from which we arrive at the value of the integration constant $C=-\frac{7}{8}c$ and the representations

(25) $$x\left(\tau_{1}\right)=a=\frac{c}{2}\left(\frac{1}{\tau_{1}}+2\tau_{1}+\tau_{1}^{3}\right),\;y\left(\tau_{1}\right)=b=\frac{c}{2}\left(\ln\frac{1}{\tau_{1}}+\tau_{1}^{2}+\frac{3}{4}\tau_{1}^{4}-\frac{7}{4}\right),$$

which are used to determine the values of c and $\tau_{1}$.

In particular, excluding the parameter c from Eq. (25), we arrive at the equation

(26) $$\beta \left(\frac{1}{\tau_{1}}+2\tau_{1}+\tau_{1}^{3}\right)=\ln\frac{1}{\tau_{1}}+\tau_{1}^{2}+\frac{3}{4}\tau_{1}^{4}-\frac{7}{4},\tau_{1}\geq 1,\beta =\frac{b}{a}$$

Eq. (26) determines the dependence $\tau_{1}\left(\beta \right)$, which can be also represented in the form

(27) $$\beta =\frac{\ln\frac{1}{\tau_{1}}+\tau_{1}^{2}+\frac{3}{4}\tau_{1}^{4}-\frac{7}{4}}{\frac{1}{\tau_{1}}+2\tau_{1}+\tau_{1}^{3}}$$

For the asymptotic values of the parameter, β≪1 and β≫1, the solutions of Eq. (26) have the following asymptotic representations

(28) $$\tau_{1}\left(\beta \right)\approx 1+\beta ,\beta \ll 1;\;\tau_{1}\left(\beta \right)\approx \frac{4}{3}\beta ,\beta \gg 1$$

The values of optimized functional for the variational problem in the formulation of Eq. (2) are obtained in accordance with the above parametric representations, i.e., Eq. (24), and Eq. (20), that is

(29) $$J_{*}\left(\beta \right)=\frac{x_{*}^{2}}{2}\left[1+\frac{1}{8}\left(\ln\;\tau_{1}+\frac{\tau_{1}^{2}-1}{4}\left(-\frac{2}{\tau_{1}^{2}}+3\tau_{1}^{2}+13\right)\right)\right]$$

where $\beta =\frac{b}{a}$ and the dependence $\tau_{1}\left(\beta \right)$ is solution of Eq. (27).

## 4 Numerical results

**Fig. 2:**
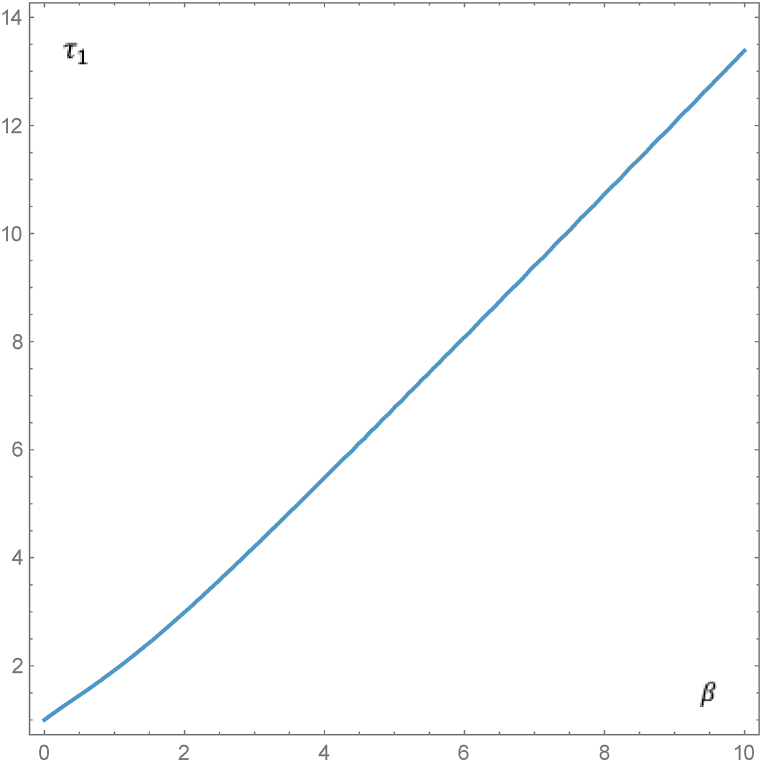
Parametric dependence $\tau_{1}\left(\beta \right)$ determined by Eq. (26) in the interval of parameter variation 0≤β≤10. The change in asymptotic behavior, as described by Eq. (28), can be seen in the lower branch of the curve.

This section presents the numerical results for dependences $\tau_{1}\left(\beta \right)$ and y(x). First of all, in order to estimate the efficiency of the asymptotic representations (28), we present the numerical solution of Eq. (26) for the parameter values of β=0.1 and β=10, where the corresponding values obtained from asymptotic equation (28) are shown in parentheses. These values are as follows: β=0.1: $\tau_{1}=$ 1.0960748 (1.1); β=10: $\tau_{1}=$ 13.3853 ($\tau_{1}=$ 13.3333). The dependence $\tau_{1}\left(\beta \right)$ determined by Eq. (26) in the interval of parameter change 0≤β≤10 is shown in Fig. 2.

The value of the corner point $x_{*}$ of the optimal solution y(x), in accordance with relations (20) and (25), is determined by the expression

$$x_{*}=\frac{4a}{\frac{1}{\tau_{1}}+2\tau_{1}+\tau_{1}^{3}}$$

and for the asymptotic values of the parameter, taking into account Eq. (28), it is described by the relations

(30) $$x_{*}\approx a\left(1-\beta \right),\beta \ll 1;\;x_{*}\approx \frac{27}{16}\frac{a}{\beta^{3}},\beta \gg 1$$

**Fig. 3:**
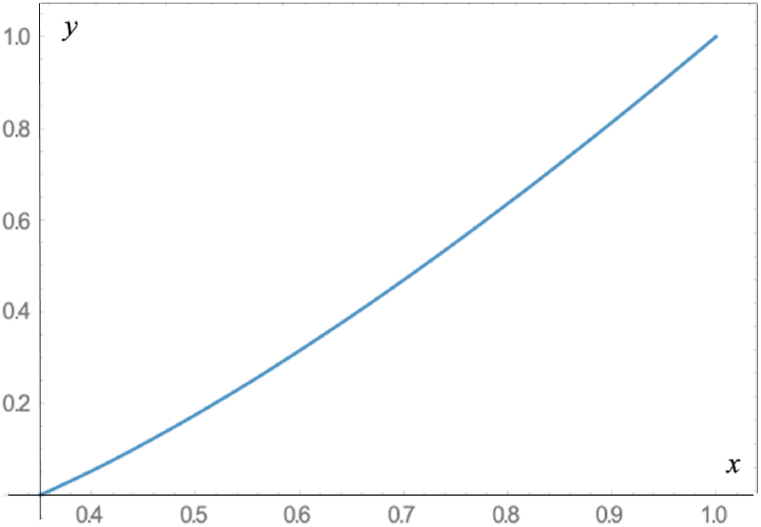
Dependence y(x) determined by Eq. (24) for $x_{*}\leq x\leq 1$. The optimal contour corresponds to the values a=b=1(β=1).

Finally, Fig. 3 shows the shape of the optimal contour y(x) corresponding to the values a=1, b=1(β=1). For these values we get $\tau_{1}=$ 1.916801245641185, $x_{*}=$ 0.3509425720484109, and $c=x_{*}/2$.

The asymptotic expressions for the values of optimized functional, that is Eq. (29), in the case of optimal contours corresponding to the asymptotic values of parameter, i.e., β≪1 and β≫1, and the value a=1 in Eqs. (28), (30) take the form

(31) $$J_{*}\left(\beta \right)\approx \frac{1}{2}\left(1-\beta \right)\;\left(\beta \ll 1\right);\;J_{*}\left(\beta \right)\approx \frac{27}{64}\frac{1}{\beta^{2}}\;\left(\beta \gg 1\right)$$

It follows from the second expression in Eq. (31) that as the ratio $\beta =\frac{b}{a}$ increases, the value of optimized functional decreases and tends to zero. From a practical point of view, this means that an axisymmetric body with a blunt head-form moving in the rarefied medium experiences less resistance.

We also give the value of functional in the formulation of Eq. (2) corresponding to the optimal contour y(x) for a=b=1(β=1), that is J[y(x)]=0.18741. To evaluate the efficiency of the obtained optimal solution, we note that the functional value is $J\left[y_{1}\left(x\right)\right]=0.25$ for the dependence $y_{1}\left(x\right)=x$, where 0≤x≤1, which corresponds to the generatrix of the lateral surface of a cone with the vertex placed at the origin of coordinates and the *x*-axis directed along the cone axis. We can also calculate the value of this functional for the contour $y_{2}\left(x\right)$ determined by the relations ${\left(x-1\right)}^{2}+y^{2}=1$, where 0≤x≤1 and y≥0, so that in the considered interval of variation of the variable *x* we have $y_{2}\left(x\right)=\sqrt{x\left(2-x\right)}$ and $y_{2}^{\prime }\left(x\right)=\frac{1-x}{\sqrt{x\left(2-x\right)}}$, which is describing a hemisphere during rotation around the *x*-axis, that is $J\left[y_{2}\left(x\right)\right]=0.41667$. One can note that $y_{1}\left(1\right)=1$ and $y_{2}\left(1\right)=1$ for both contours $y_{1}\left(x\right)$ and $y_{2}\left(x\right)$, which corresponds to the value of β=1. In passing, one should further note that the models for cone and convex hemisphere are important as these types of surfaces form the structural elements of the real flying objects (see, for example, Ref. [12]).

## 5 Conclusions

In this paper, we present the solution of the Newton's aerodynamic variational problem characterized by the presence of unilateral constraints in its mathematical formulation. This property does not allow the use of classical methods for solving variational problems, and, in connection with this, the methods of the optimal control theory have been recently applied to solve the Newton's aerodynamic problem. Transformation of the Newton's aerodynamic variational problem with unilateral constraints to the classical isoperimetric variational problem is given in this paper, followed by the exact solution of this problem. The obtained solution coincides with the solution given in Ref. [3] by transforming the variational problem in the formulation of Eq. (2) to the optimal control problem. Coincidence of these solutions confirms the correctness and efficiency of the method adopted in the article for solving this variational problem. The value of the optimized functional for the optimal contour is given. Using standard procedures of numerical computation, e.g., MATLAB (MathWorks) or Wolfram Mathematica, is extremely efficient in verifying numerical results, while analytical methods are desirable when it is possible to obtain exact formulas. Finally, to evaluate the efficiency of the optimal solution, the values of the functional for cone and hemisphere are also given. From the comparison of these numerical results with the optimal contour value it follows an increase of their resistance as the ratio tends to 1.33 for cone and 2.22 for hemisphere, i.e., the optimization effect is significant.

## Author contribution statement

Alexandr A. Barsuk: Conceived and designed the experiments; Performed the experiments; Analyzed and interpreted the data; Wrote the paper.

Florentin Paladi: Conceived and designed the experiments; Performed the experiments; Analyzed and interpreted the data; Contributed reagents, materials, analysis tools or data; Wrote the paper.

## Data availability statement

Data will be made available on request.

## Declaration of competing interest

The authors declare that they have no known competing financial interests or personal relationships that could have appeared to influence the work reported in this paper.
